# Tranexamic Acid versus Placebo to Prevent Blood Transfusion during Radical Cystectomy for Bladder Cancer (TACT): Study Protocol for a Randomized Controlled Trial

**DOI:** 10.1186/s13063-018-2626-3

**Published:** 2018-05-02

**Authors:** Rodney H. Breau, Luke T. Lavallée, Sonya Cnossen, Kelsey Witiuk, Ilias Cagiannos, Franco Momoli, Gregory Bryson, Salmaan Kanji, Christopher Morash, Alexis Turgeon, Ryan Zarychanski, Ranjeeta Mallick, Greg Knoll, Dean A. Fergusson

**Affiliations:** 1 Ottawa Hospital Research Institute, Ottawa, ON Canada; 2 Division of Urology, Department of Surgery, The Ottawa Hospital, Ottawa, ON Canada; 3 Department of Anesthesiology and Pain Medicine, University of Ottawa and Ottawa Hospital, Ottawa, ON Canada; 4 CHU de Québec, Université Laval, Québec City, QC Canada; 5 Department of Internal Medicine, Section of Medical Oncology and Haematology, University of Manitoba, Winnipeg, MB Canada

**Keywords:** Bladder cancer, Radical cystectomy, Tranexamic acid, Blood transfusion

## Abstract

**Background:**

Radical cystectomy for bladder cancer is associated with a high risk of needing red blood cell transfusion. Tranexamic acid reduces blood loss during cardiac and orthopedic surgery, but no study has yet evaluated tranexamic acid use during cystectomy.

**Methods:**

A randomized, double-blind (surgeon-, anesthesiologist-, patient-, data-monitor-blinded), placebo-controlled trial of tranexamic acid during cystectomy was initiated in June 2013. Prior to incision, the intervention arm participants receive a 10 mg/kg loading dose of intravenously administered tranexamic acid, followed by a 5 mg/kg/h maintenance infusion. In the control arm, the patient receives an identical volume of normal saline that is indistinguishable from the intervention. The primary outcome is any blood transfusion from the start of surgery up to 30 days post operative. There are no strict criteria to mandate the transfusion of blood products. The decision to transfuse is entirely at the discretion of the treating physicians who are blinded to patient allocation. Physicians are allowed to utilize all resources to make transfusion decisions, including serum hemoglobin concentration and vital signs. To date, 147 patients of a planned 354 have been randomized to the study.

**Discussion:**

This protocol reviews pertinent data relating to blood transfusion during radical cystectomy, highlighting the need to identify methods for reducing blood loss and preventing transfusion in patients receiving radical cystectomy. It explains the clinical rationale for using tranexamic acid to reduce blood loss during cystectomy, and outlines the study methods of our ongoing randomized controlled trial.

**Trial registrations:**

Canadian Institute for Health Research (CIHR) Protocol: MOP-342559; ClinicalTrials.gov, ID: NCT01869413. Registered on 5 June 2013.

**Electronic supplementary material:**

The online version of this article (10.1186/s13063-018-2626-3) contains supplementary material, which is available to authorized users.

## Background

Bladder cancer is the sixth most common cancer in North America with an estimated 80,000 new diagnoses in 2011 [1, 2]. Among incident cancers it is estimated that 8% (6400 per year) will undergo radical cystectomy [3]. Radical cystectomy is an advanced surgical procedure that involves removal of the urinary bladder, adjacent organs, and pelvic lymph nodes. Removal of the urinary bladder also necessitates urinary reconstruction using an isolated segment of small or large intestine for urinary diversion. The extensive extirpative and reconstructive components of this surgery result in prolonged operative times and are associated with significant risk of complications including high risk of perioperative bleeding and subsequent need for blood transfusion [4]. Among individual surgeons at institutions that perform many procedures, median intraoperative blood loss is between 600 and 1700 mL [5–7]. The incidence of at least one intraoperative blood transfusion is 9 to 67% [5–9], and the postoperative transfusion risk is at least 15% [6, 9]. Among cystectomy patients who receive transfusion, a median of 2 units of blood cells are given [6, 9].

Lysine analog drugs are synthetic derivatives of the amino acid lysine that reversibly block lysine-binding sites on plasminogen molecules [10]. This action prevents the conversion of plasminogen to plasmin, the active enzyme that degrades fibrin clots. Therefore, lysine analogs decrease the breakdown of clots and are considered anti-fibrinolytics. There are two commonly studied lysine analogs, tranexamic acid and epsilon-aminocaproic acid. Both of these drugs have been shown to decrease blood transfusion need during some surgeries without a significant increase in adverse events.

In 2011, urologists who specialize in cancer treatment (members of the Society of Urologic Oncology) were surveyed about the methods they used to prevent blood loss during radical cystectomy [11]. Respondents were asked about their use of systematic techniques, systemic drugs, and topical agents to reduce blood loss and prevent blood transfusion. Topical agents were commonly used among expert urologists (73% used oxidized cellulose polymer; 53% used gelatin and thrombin matrix) and 28% used at least one systematic technique such as autologous blood recovery. Systemic drugs, such as lysine analogs, were used by only 5% of surgeons.

The infrequent use of lysine analogs during radical cystectomy may be due to lack of high-quality evidence supporting their effectiveness in this setting. While numerous cardiac and orthopedic surgery studies have evaluated the effect of intraoperative lysine analogs [12], a recent systematic review of major pelvic surgery failed to identify a single cystectomy trial [13].

In summary, radical cystectomy is associated with a high risk of clinically significant blood loss and blood transfusion, while lysine analogs have been shown to decrease these risks during other types of surgery. Therefore, our objective is to determine the effect of lysine analogs during radical cystectomy using a placebo-controlled randomized trial. We chose to use tranexamic acid as the study drug because of its ease of administration, demonstrated benefit in other procedures, safety profile, and familiarity of use in other surgical procedures.

### Objectives and research questions

The primary objective is whether the use of systemic tranexamic acid compared to placebo reduces the proportion of radical cystectomy patients requiring red blood cell transfusion up to 30 days post operative (anticipated 50% transfusion rate with placebo to 35% with tranexamic acid). Secondary objectives are to determine the effect of tranexamic acid on intraoperative blood loss, number of transfused blood products, and post-operative complications. Specifically, thrombotic events will be evaluated.

## Methods

### Study setting and design

This study was initiated at The Ottawa Hospital, a regional cancer referral center in Ottawa, Canada. Thirteen urologic oncology centers in Canada have committed to participate (University of Ottawa, University of Toronto, University of Western Ontario, Université de Montréal, McGill University, Université Laval, Dalhousie University, Queen’s University, McMaster University (Hamilton Health and St. Joseph’s Healthcare), University of Alberta, University of British Columbia, and University of Manitoba). Patients are randomized to receive either tranexamic acid (intervention) or placebo (control).

**Fig. 1:**
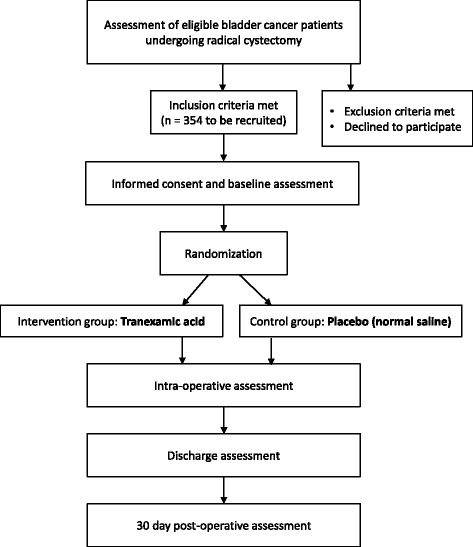
Study flowchart

**Fig. 2:**
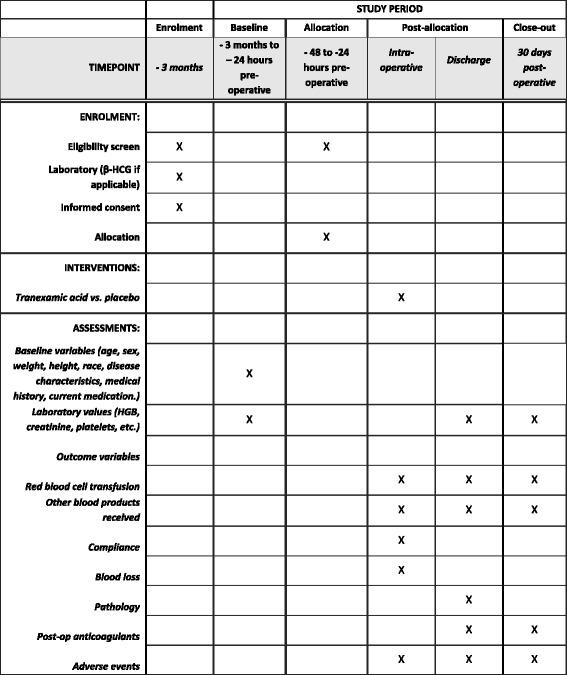
Timeline of study procedures and outcome assessments

In keeping with a pragmatic approach to this study, we have adopted permissive eligibility criteria, an easily administered protocol, outcomes that are of relevance to physicians, patients, and health administrators, and limited data collection. Evaluation and flow of patients prior to, during, and following surgery will be according to usual standard of care. The study flowchart is presented in Fig. 1, and the timeline of study/outcome assessments is outlined in Fig. 2.

### Eligibility criteria

#### Study population

Patients undergoing radical cystectomy for treatment of bladder cancer. In keeping with a pragmatic approach to this study, eligibility criteria are inclusive, allowing for the wide generalizability of results.

#### Inclusion criteria

Consenting adult patients (aged ≥ 18 years) undergoing radical cystectomy for bladder cancer are considered eligible, regardless of tumor stage and histology. Patients who have undergone previous surgery, radiation, or chemotherapy may be included. Extent of lymph node dissection is not prescribed. All forms of urinary diversion (conduit, orthotopic continent, or continent cutaneous) are allowed. Use of perioperative thromboprophylaxis (e.g., anti-coagulants, thromboembolism-deterrent stockings, sequential compression devices) are permitted and are at the discretion of the care providers.

#### Exclusion criteria

Patients are excluded from enrollment if they (1) are unable or unwilling to receive red blood cell products due to medical or personal reasons; (2) are pregnant (confirmed by βHCG test); (3) have a known allergy to tranexamic acid; (4) have acquired disturbances of color vision; (5) have a history of subarachnoid hemorrhage; or (6) have thromboembolic disease (active or diagnosed within 1 year), such as deep vein thrombosis (DVT), pulmonary embolism (PE), or cerebral thrombosis.

### Study interventions

The study intervention is Canadian-sourced tranexamic acid (Cyklokapron® made by Pfizer). Tranexamic acid is administered intravenously at a dose of 10 mg/kg within 10 min (loading dose) prior to the surgical incision, followed by 5 mg/kg/h continuous maintenance infusion for the length of surgery (stopped ± 20 min from skin closure). For example, an 80-kg patient would receive 800 mg prior to incision and a 400 mg/h infusion for the duration of surgery.

#### Description and justification of dose

There is no defined dosing schedule for tranexamic acid administration. A wide range of doses and schedules have been used in clinical trials [12]. In 34 cardiac surgery trials investigating the efficacy of tranexamic acid, the loading dose ranged from 2.5 to 100 mg/kg while the maintenance infusions ranged from 0.25–16 mg/kg/h [12]. In 31 non-cardiac surgery trials investigating the efficacy of tranexamic acid, the majority (27) in orthopedic surgery, loading doses ranged from 10 to 40 mg/kg and maintenance infusions ranged from 1 to 40 mg/kg/h [12]. The most common loading dose from all trials was 10 mg/kg and the most common infusion dose was 10 mg/kg/h in non-cardiac surgery and 1 mg/kg/h in cardiac surgery (not including doses of drug used to prime the bypass machine). Dosing strategies were highly variable as some studies did not use a loading dose while others gave only single or intermittent doses.

The serum half-life of tranexamic acid is 120 min when administered intravenously [14]. Without a loading dose it would take as long as 10 h to reach a steady-state serum concentration when the maximum treatment effect would be observed. Therefore, we decided that a loading dose is essential to ensure adequate drug concentration at the time of the initial incision so that the maximum effect of the drug coincides with the greatest risk for bleeding. Furthermore, given the relatively short half-life of tranexamic acid and the long duration of cystectomy surgery, a maintenance infusion is more likely to maintain therapeutic serum concentrations compared to intermittent bolus dosing.

Given the safety profile of tranexamic acid and the potential dose-related response (in vitro) we believed that it was important to choose a dose large enough to be confident that we are achieving therapeutic drug concentrations. A 10 mg/kg loading dose has been frequently studied and should achieve a therapeutic serum concentration. In addition, the maintenance infusion dose suggested for bypass surgery (5 mg/kg/h) is within the range of studied doses and should be sufficient to maintain drug levels throughout surgery.

#### Pharmacy preparation and administration of tranexamic acid or placebo

Ampoules of tranexamic acid (10 mL) of concentration 100 mg/mL are secured by the pharmacy. Mixtures of tranexamic acid remain stable for up to 24 h when refrigerated; therefore, the local research pharmacist or pharmacy technician accesses the online patient allocation and prepares the study drug or placebo within a 24 h period prior to the anticipated completion of the scheduled cystectomy. All mixtures are kept refrigerated from the time of mixing until time of use unless used immediately. The weight-based dose to administer for each patient is confirmed by the administering anesthesiologist using a weight-based dosing nomogram provided by the coordinating center’s pharmacy research technician. The research site’s pharmacy staff also has access to the patient’s weight and dosing nomogram in order to prepare the study products. Study drugs are stored in a secure, locked location at each site’s pharmacy department. Health Canada regulations mandate monitoring and documenting temperature daily as the medication must be stored at room temperature prior to mixing.

#### Placebo control

As there is no standard of care concerning administration of anti-fibrinolytic agents in cystectomy procedures, controls follow the same dosing and schedule described above, but receive 0.9% sodium chloride only.

Other techniques, agents, or equipment may be used at the discretion of the anesthesiologist/surgeon to prevent blood loss, and are recorded. These may include acute normovolemic hemodilution, autologous blood banking, systemic hemostatics (e.g., factor VIIa), topical agents (e.g., oxidized cellulose), and instruments such as thermal devices (e.g., Ligasure®).

### Study outcomes

#### Primary outcome

The primary outcome of the trial is whether or not the patient was transfused at least 1 unit of packed red blood cells up to 30 days post operative. We will compare the proportion transfused at least 1 unit of packed red blood cells between tranexamic acid and placebo study arms. In keeping with our pragmatic approach and difficulty in defining a transfusion protocol, there are no strict criteria to mandate the transfusion of blood products. The decision to transfuse is entirely at the discretion of the treating physicians who are blinded to patient allocation. Physicians are allowed to utilize all resources to make transfusion decisions, including serum hemoglobin concentration and vital signs.

#### Secondary outcomes

Secondary outcomes will assess other transfusion metrics and perioperative safety, cumulative to 30 days post operative. Documentation includes total units of red blood cells transfused, occurrence of postoperative bleeding requiring intervention (e.g., reoperation or angioinfarction), occurrence of platelet transfusion, total units of platelets transfused, occurrence of plasma transfusion, total units of plasma transfused, estimated intraoperative blood loss, and change in hemoglobin. Safety outcomes include occurrence of severe adverse events, such as DVT, PE, myocardial infarction (MI), and cerebral infarction. We also capture treatment “failures” noted as the need for anti-hemorrhagic rescue interventions such as topical agents (e.g., oxidized cellulose) and recombinant factor VIIa.

#### Sequence and frequency of follow-up

Data is collected prior to surgery, during surgery, at the time of hospital discharge, and at 30 days post operative (Fig. 2). At each data collection time point, a detailed review of the health record is conducted by the site research coordinator. Laboratory tests are drawn as per standard clinical care at each institution. A 30-day post-surgery follow-up for adverse events is conducted through both health records and a 30-day phone interview. If the patient reports re-admission to hospital or outpatient intervention for a complication within 30 days of surgery, the medical record from the outpatient visits is reviewed and abstracted. Case Report Forms (CRFs) are completed at each study time point and are supported fully by source documents (charts, reports, laboratory result sheets).

### Sample size

A patient record review from The Ottawa Hospital indicated that red blood cell transfusions were performed in approximately 60% of patients with bladder cancer undergoing radical cystectomy. However, we anticipate that transfusion rates are likely to decrease slightly while under the study protocol. From the results of our survey, systemic anti-fibrinolytics are rarely used during radical cystectomies; to influence practice we suggest that tranexamic acid will have to produce a substantial reduction in the proportion of patients requiring transfusion. To assist in determining the minimally important effect of tranexamic acid deemed necessary to change clinical practice, Canadian experts in the surgical treatment of bladder cancer were surveyed in February 2012. Minimally important effect sizes ranged from an absolute difference of 15% (relative risk (RR) 30%) to 30% (RR 60%) and are consistent with effects observed in many clinical trials of tranexamic acid [12]. From our systematic review of lysine analogs to prevent bleeding during pelvic surgery, the pooled estimate favored lysine analogs (RR 0.69; 95% confidence interval (CI) 0.47 to 1.00) [13].

If 50% of placebo patients will require at least one transfusion within 30 days of surgery, and if the use of tranexamic acid reduces the need for transfusion within 30 days to 35% of patients (15% absolute reduction), and assuming 80% power to detect this absolute difference, with a two-sided alpha level of 5%, the required sample size is 340 people (170 per treatment group). To account for 2% non-compliance with the protocol (possibly due to intraoperative complications or protocol deviations) the total sample size required was estimated to be 354 patients (177 per arm) [15].

### Recruitment

We are implementing a number of initiatives to maintain recruitment, including regular tracking, monitoring and reporting of recruitment activity at each participating site, regular newsletters, progress reports and regularly scheduled investigator and research staff meetings. Participants have the right to withdraw from the study at any time, but are advised to discuss this decision with the site’s qualified investigator or study coordinator before stopping the study. Information given to the research team before the participant withdraws consent may still be used to analyze the study.

### Allocation of intervention

#### Randomization

Eligible and consenting patients are randomized using computer-generated random numbers before surgery in a 1:1 allocation ratio to one of the two arms of the study using permuted blocks of variable length (2, 4, and 6). Patients are also stratified by center.

### Blinding

#### Blinding of allocation arm

Randomization, centralized allocation management, and permuted blocks of variable length are used to ensure that participating centers and physicians remain unaware of the randomization sequence. The pharmacy research staff have exclusive access to their site’s randomization schedule and are expressly forbidden to discuss individual treatment allocation with the study team, the patient, the operating room and clinical care team unless emergency un-blinding is requested and approved by the principle investigator.

#### Blinding of intervention

Tranexamic acid, diluted in saline or undiluted, is clear, colorless, and indistinguishable from saline both at rest and when vigorously agitated [16]. Matching placebo maintenance infusions are identical saline bags without discarded saline or added drug in order to maintain the same volume and weight. To minimize sources of selection and ascertainment biases, anesthesiologists, surgeons, investigators, research staff, and members of the Data Safety and Monitoring Board (DSMB) are all blinded to randomization schemes and treatments administered; only the statistician has access to randomization schemes. The research pharmacist does not have contact with the study team or the patient, and is expressly forbidden to discuss individual treatment allocation with the operating room and clinical care team (a signed confidentiality agreement is obtained from each site research pharmacy personnel). Clinical teams are permitted to unmask treatment allocation only under exceptional circumstances. A request to unmask allocation for an individual patient is made to the coordinating center. A clinical member of the Executive Committee (24 h availability) reviews the request to ensure that it is necessary, based on pre-established criteria.

### Trial management

The Ottawa Methods Center of the Ottawa Hospital Research Institute is responsible for coordination and day-to-day management of the trial, where the investigators have extensive experience of conducting multi-center trials. The data management services group is responsible for initial database design and setup, web-based online enrollment, and randomization, electronic data-form maintenance, and data storage. About 20% of documents at each site are reviewed for data quality. Each site has a site investigator and at least one research coordinator dedicated to this project. The site research coordinator has the responsibility to verify patient eligibility, seek informed consent, attend procedures and randomize patients via the online website, accurately fill out all data collection forms, and report any serious adverse events (SAEs).

### Statistical methods

Patient baseline characteristics will be summarized with descriptive statistics. Comparative analyses will be based on an intention-to-treat principle (i.e., based on the participant’s randomized study arm allocation). To address the primary objective of the effect of systemic tranexamic acid compared to placebo on the proportion of patients requiring red blood cell transfusion within 30 days, a chi-square test of proportions will be used. The effect size will be derived with an unadjusted RR and 95% confidence limits. If notable baseline imbalances are present, an adjusted estimate will be derived for the RR by stratifying on the potential confounders and pooling with a Mantel-Haenszel RR estimator. Secondary analyses of the effect of tranexamic acid on the volume of intraoperative blood loss and the perioperative adverse event rate will follow in a similar fashion, using chi-square or *t* tests, depending the outcome, and logistic or linear regression to estimate the magnitude of effect. A Poisson regression model will be fit to assess the difference in the number of transfused blood products between trial arms. Subgroup analyses will also be presented for clinically important baseline factors that may influence the effect of the drug, including sex, patient age (< 60 years, 60–70 years, 71–80 years, and < 80 years), preoperative hemoglobin (< 100 g/L, 100–120 g/L, and > 120 g/L), and previous history of cardiac disease (yes/no). Data is compiled by the study statistician and reviewed by an independent DSMB every 6 months. No interim analysis is planned for this trial.

### Data monitoring

#### Steering Committee and Data Safety Monitoring Board

All collaborators, site investigators, and research staff serve on the Trial Steering Committee. The committee aims to meet via teleconference every 6 months.

The DSMB has full responsibility for the monitoring of response variables for adverse events throughout the trial. This committee functions independently from all other study committees and serves in an advisory role to the Executive Committee. The DSMB consists of three individuals, covering requisite expertise in clinical epidemiology, biostatistics, urologic oncology, and anesthesia, and may consult external experts regarding specific outcome evaluations.

#### Stopping rules

No early stopping rules have been developed for this trial; early stopping criteria are at the discretion of the members of the independent DSMB. The DSMB may make recommendations to terminate or continue the study. Early stopping of the study will only be based on appraisal of SAEs and will be at the discretion of the clinical experts of the independent DSMB.

#### Individual discontinuation criteria

In the event of an anaphylactic reaction to the allocated agent, the attending anesthesiologist will stop the intervention and will treat the anaphylaxis without un-blinding the allocated agent.

#### Harms

Adverse events are documented as part of the CRFs. Severe adverse events are submitted to the central coordinating center using a study-specific reporting form. We do not expect any issues with safety, thus no formal interim analyses and stopping rules have been planned; however, having built-in independent safety oversight is prudent. Adverse events are reported to Health Canada as per International Conference on Harmonization/Good Clinical Practice (ICH-GCP) and Health Canada Division 5 regulations.

#### Summary of the known and potential risks and benefits to participants involved in the trial

Smoking is a common risk factor for bladder cancer and cardiovascular disease, thus many bladder cancer patients are at risk for thromboembolic complications such as MI, stroke, DVT, and PE. As outlined, given the mechanism of action of tranexamic acid, that being the inhibition of clot breakdown, it could be anticipated that this medication would increase the risk of thromboembolic events. Contrary to this notion, previous studies of tranexamic acid with enrollment of thousands of patients with cardiovascular disease have not observed an increased risk of these adverse events [12].

Tranexamic acid has been approved for use by Health Canada and has a well-established side-effect profile [17]. Tranexamic acid is used predominantly in cardiac surgery, but has also been studied in orthopedic surgery and trauma patients [18]. A recently updated systematic review of lysine analogs (including tranexamic acid) identified 252 trials, of which 173 were conducted in cardiac surgery and 53 in orthopedic surgery [12]. Data regarding adverse effects of tranexamic acid was drawn from this Cochrane systematic review as well as a recent randomized controlled trial (RCT) examining the use of tranexamic acid in a trauma setting [18]. The Cochrane review of anti-fibrinolytics identified 65 trials containing 2528 patients comparing tranexamic acid with placebo to prevent blood loss and transfusion [12]. However, none of the trials in this systematic review included patients treated with radical cystectomy. From this review of RCTs, the risk of red blood cell transfusion was 39% lower among patients who received tranexamic acid compared to placebo or standard of care (RR 0.61; 95% CI 0.53 to 0.70).

The use of tranexamic acid was not associated with increased risk of death (RR 0.60, 95% CI 0.33 to 1.10), MI (RR 0.79, 95% CI 0.41 to 1.52), or stroke (RR 1.23, 95%CI 0.49 to 3.07). Tranexamic acid was not associated with increased risk of developing a DVT (RR 0.71, 95% CI 0.35 to 1.43) or a PE (RR 0.67, 95% CI 0.23 to 1.99). Treatment with tranexamic acid did not appear to increase the risk of developing renal failure or dysfunction (RR 0.89, 95% CI 0.33 to 2.37). The CRASH-2 trial randomized 20,211 adult trauma patients to tranexamic acid or placebo [18]. In this trial, there were 33 deaths (0.3%) in the tranexamic acid arm vs. 48 (0.5%) in the placebo arm related to vascular occlusion (*p* = 0.96) including 7 vs. 22 deaths from MI, 8 vs. 5 from stroke, and 18 vs. 21 from PE, respectively. Vascular occlusive events, fatal or non-fatal, did not differ significantly with 168 (1.7%) patients with one or more vascular occlusive events (MI, stroke, PE, DVT) in patients receiving tranexamic acid vs. 201 (2%) in patients receiving placebo (RR 0.84, 95% CI 0.68 to 1.02).

In a retrospective study of 604 patients undergoing cardiac surgery [19], it was reported that post-operative seizures occurred significantly more frequently in patients treated with tranexamic acid compared to epsilon-aminocaproic acid. Twenty-one tranexamic acid-treated patients (7.6%) compared to 13 (3.3%) epsilon-aminocaproic acid-treated patients (*p* = 0.019) developed post-operative seizures. There was no control group in this study to which comparisons could be made. Patients in this study received a high dose of tranexamic acid with an initial 2-g bolus at the beginning of cardiopulmonary bypass followed by infusion of 0.5 g/h until chest closure and 2 g added to the priming fluid of the cardiopulmonary bypass system. Other recent retrospective studies have also shown that tranexamic acid, when given in high doses of 61 to 259 mg/kg, is associated with elevated rates of post-operative seizure compared to epsilon-aminocaproic acid [20, 21]. We have not found data indicating increased risk of seizure in trials using a similar dose to what we have chosen for our trial.

Finally, the recent tranexamic acid RCT of 200 radical prostatectomy patients used a comparable dose to our study (500 mg loading and 250 mg/h infusion). They captured adverse events at 1 and 6 months following surgery [22]. No deaths occurred and thromboembolic events were more common in the control group (five in the placebo vs. two in the tranexamic acid arm; RR 0.4; 95%CI 0.09 to 1.74).

Given the effectiveness of tranexamic acid in reducing blood loss during other surgical procedures without increasing the number of thromboembolic events compared to placebo, we hypothesize that tranexamic acid will eliminate the need for transfusion in a significant proportion of patients, reduce overall blood utilization, and not result in increased incidence of adverse events. Despite the large volume of evidence establishing safety of tranexamic acid in cardiac and orthopedic surgical patients, we acknowledge the theoretical risks and, therefore, we are monitoring vascular occlusive events, seizures, and other potential serious adverse reactions.

### Auditing

#### Compliance with study allocation

In this study, compliance refers only to receiving the study intervention administered during the intraoperative period, under supervision by attending urologists and anesthesiologists. We classify patients as being fully compliant if the patient is administered a complete dose schedule of the allocated agent (tranexamic acid or placebo), partially compliant if the patient is administered an incomplete dose schedule of the allocated agent, and non-compliant if the patient does not receive the allocated agent. Reasons for partial and non-compliance may range from errors in randomization/drug administration, physician and pharmacy errors in allocation, intraoperative complications, death, etc. Compliance is not based on patient tolerance and is very likely to be random. We expect partial/non-compliance to be less than 1%, although our sample size adjustment accounts for a total of 2% partial compliance and non-compliance. The degree of compliance is documented in the intraoperative CRF. Any partial compliance or non-compliance requires additional documentation by way of the Protocol Deviation or Protocol Violation Form.

#### Loss to follow-up

We expect complete follow-up at hospital discharge and at 30 days postoperative given the complexity of care and necessary short-term follow-up. From past experience in a high-risk cardiac population [23] and given the captive patient population, we expect greater than 95% follow-up at 1 month. In a recently published trial of radical prostatectomy patients, all 200 patients completed follow-up [22].

### Monitoring procedures

Study monitoring is a sponsor responsibility as outlined in ICH-GCP. This responsibility has been delegated back to the qualified investigator as per N2 standard operating procedure (SOP) 901.001.

The qualified investigator assigns a suitably trained monitor to perform study monitoring at all participating sites. The monitor is not directly involved in the study. The monitor is trained on ICH-GCP, Health Canada Division 5 Regulations, Tri-Council Policy Statement: Ethical Conduct for Research Involving Humans (TCPS2), study protocol, OHRI SOPs, and study-specific procedures.

#### Site monitoring

The trial data, compliance, and adverse events are rigorously monitored using both remote and on-site methods of surveillance. This ensures that trial-related data is accurate, complete, and verifiable from source documents and that participant rights and safety are protected. The monitor verifies compliance with the regulatory requirements, protocol, GCP, study-specific procedures, and participant eligibility. In addition to evaluating the reported data for accuracy and completeness, the monitor identifies any trends in data that may be indicative of insufficient documentation or protocol deviations. Discrepancies noted in the data are recorded and the site is informed of all observations in the subsequent monitoring report.

The site monitor addresses deficiencies to the appropriate study team member in order to implement corrective actions or to recommend follow-up procedures. All observations noted during the monitoring visit appear in the monitoring report. The monitor assesses study files and documentation against ICH-GCP, regulatory requirements, protocol, OHRI SOPs and any study-specific SOPs.

#### Interim monitoring visits

To ensure patient safety and data integrity, regular remote monitoring visits are performed for each study site. If remote monitoring shows discrepancies in data or lack of compliance with regulations, or if requested by the DSMB or the site investigator, on-site monitoring is performed. The study monitor assesses: CRF source data verification (SDV), patient eligibility and consent, study-specific SOPs, delegation logs, SAEs for recording and reporting completeness, regulatory documentation (for site and/or sponsor), training documents, protocol-defined endpoints, essential document maintenance, deviation/violation recording and reporting, drug accountability, privacy considerations, and any protocol-specific procedures.

For remote monitoring, de-identified data from each site is sent to the monitor 6 months after site activation via secured courier. All data to be couriered is checked by the site coordinator prior to sending in order to ensure that patient privacy and confidentiality is maintained. No identifying materials are sent off-site. Documentation sent to the monitor for remote SDV includes: copies of signed and dated de-identified laboratory assessments, copies of signed and dated de-identified patient assessment forms (CRFs) with corresponding de-identified source documents, copies of de-identified source documentation that supports subjects’ eligibility to be enrolled into the study, copies of de-identified Investigator progress notes regarding patient-related decisions, signed and dated training logs as well as copies of materials used to train study staff (slide presentations, hand-outs).

### Dissemination plan

Once the trial is completed, results will be presented at an international research meeting and published in a peer-reviewed journal.

## Discussion

Blood loss resulting in transfusion is common during radical cystectomy. As shown in previous studies, tranexamic acid has the potential to significantly reduce operative blood loss. However, based on a recent survey of urologists and a recent systematic review of the academic literature, tranexamic acid is not commonly used by urologists and no previous trials have assessed the benefits and harms of this drug for patients treated with radical cystectomy. To the best of our knowledge, this is the only registered trial evaluating a lysine analog during cystectomy (ClinicalTrials.gov search on 7 July 2017).

Use of tranexamic acid during surgery at the study dose is inexpensive (less than 40 Canadian dollars per surgical case), technically easy to administer, and has been successfully performed in several trials evaluating other surgical procedures. Anesthesiologists are familiar with the use of tranexamic acid during cardiac surgery, orthopedic surgery, and other procedures where significant blood loss is anticipated. However, the uncritical use of tranexamic acid is not warranted without rigorous and definitive evidence to inform clinical practice. While most individual studies of tranexamic acid for non-urological surgeries revealed benefit of the drug, some did not. For example, in a study of myomectomy (surgical removal of benign tumors from the uterus), 50 patients were randomized to tranexamic acid and 50 were randomized to a control group which did not receive tranexamic acid [24]. In this study, tranexamic acid did not reduce the risk of receiving an allogeneic blood transfusion (RR 1.50, 95% CI 0.75 to 3.01). This evidence conflicts with findings that tranexamic acid was efficacious in reducing the number of transfusions required in other pelvic surgeries (e.g., radical prostatectomy) [22] and further highlights the need for more in-depth investigation of the effectiveness of tranexamic acid in pelvic surgery through a RCT. Furthermore, cystectomy patients are at high risk of thromboembolic complications and, theoretically, lysine analogs may increase this risk.

At the completion of the trial, we will better understand the benefits and risks of systemic tranexamic acid during radical cystectomy. Specifically, we will know if tranexamic acid reduces the risk of exposure to blood transfusion among patients undergoing radical cystectomy. Given that there are few exclusion criteria and multi-institutional involvement, we expect our findings to have broad applicability. As this is the first RCT evaluating the use of a systemic anti-fibrinolytic during radical cystectomy, the findings may have an immediate impact on patient care. If the study is negative, we can avoid unnecessary exposure of patients to tranexamic acid during radical cystectomy. If the study reveals a benefit of tranexamic acid, we will change our standard of practice regarding radical cystectomy procedures, and procedures common to other major pelvic surgeries associated with significant blood loss will be challenged (Additional file 1).

## Trial status

Since the trial opened in June 2013, 147 patients have been accrued and successfully randomized. 13 research sites are participating in the trial, eight of which are currently active. The remaining five sites are in the process of submitting their regulatory documents before site setup can begin. To date, no SAEs have been reported that were considered related to the study drug.

## Additional file

Additional file 1: SPIRIT 2013 Checklist: Recommended items to address in a clinical trial protocol and related documents. (DOCX 60 kb)

## Abbreviations

CI: Confidence interval
CRF: Case Report Form
DSMB: Data Safety and Monitoring Board
DVT: Deep vein thrombosis
hCG: Human chorionic gonadotropin
IHC-GCP: International Conference on Harmonization/Good Clinical Practice
MI: Myocardial infarction
OHRI: Ottawa Hospital Research Institute
PE: Pulmonary embolism
RCT: Randomized controlled trial
RR: Relative risk
SAE: Serious adverse event
SDV: Source data verification
SOP: Standard operating procedure
TCPS2: Tri-Council Policy Statement: Ethical Conduct for Research Involving Humans
